# Efficient designs for three-sequence stepped wedge trials with continuous recruitment

**DOI:** 10.1177/17407745241251780

**Published:** 2024-05-21

**Authors:** Richard Hooper, Olivier Quintin, Jessica Kasza

**Affiliations:** 1 Wolfson Institute of Population Health, Queen Mary University of London, London, UK; 2 School of Public Health and Preventive Medicine, Monash University, Melbourne, VIC, Australia

**Keywords:** Stepped wedge trial, optimal design, continuous recruitment, centrosymmetry, decaying intra-cluster correlation

## Abstract

**Background/Aims:**

The standard approach to designing stepped wedge trials that recruit participants in a continuous stream is to divide time into periods of equal length. But the choice of design in such cases is infinitely more flexible: each cluster could cross from the control to the intervention at any point on the continuous time-scale. We consider the case of a stepped wedge design with clusters randomised to just three sequences (designs with small numbers of sequences may be preferred for their simplicity and practicality) and investigate the choice of design that minimises the variance of the treatment effect estimator under different assumptions about the intra-cluster correlation.

**Methods:**

We make some simplifying assumptions in order to calculate the variance: in particular that we recruit the same number of participants, m , from each cluster over the course of the trial, and that participants present at regularly spaced intervals. We consider an intra-cluster correlation that decays exponentially with separation in time between the presentation of two individuals from the same cluster, from a value of ρ for two individuals who present at the same time, to a value of ρτ for individuals presenting at the start and end of the trial recruitment interval. We restrict attention to three-sequence designs with centrosymmetry – the property that if we reverse time and swap the intervention and control conditions then the design looks the same. We obtain an expression for the variance of the treatment effect estimator adjusted for effects of time, using methods for generalised least squares estimation, and we evaluate this expression numerically for different designs, and for different parameter values.

**Results:**

There is a two-dimensional space of possible three-sequence, centrosymmetric stepped wedge designs with continuous recruitment. The variance of the treatment effect estimator for given ρ and τ can be plotted as a contour map over this space. The shape of this variance surface depends on τ and on the parameter mρ/(1−ρ) , but typically indicates a broad, flat region of close-to-optimal designs. The ‘standard’ design with equally spaced periods and 1:1:1 allocation rarely performs well, however.

**Conclusions:**

In many different settings, a relatively simple design can be found (e.g. one based on simple fractions) that offers close-to-optimal efficiency in that setting. There may also be designs that are robustly efficient over a wide range of settings. Contour maps of the kind we illustrate can help guide this choice. If efficiency is offered as one of the justifications for using a stepped wedge design, then it is worth designing with optimal efficiency in mind.

## Background/aims

Stepped wedge trials are longitudinal cluster randomised trials where clusters are randomised, not to treatment conditions, but to *sequences* which dictate when each cluster will cross over uni-directionally from the control condition to the intervention condition.^1,2^ Since the seminal discussion of stepped wedge trials by Hussey and Hughes,^3^ methodological work has tended to treat prospective time as a series of discrete periods, and we now understand a great deal about the optimal design of this kind of stepped wedge trial.^4–9^

But in many stepped wedge trials, including the first to be published with this label,^10^ participants from a cluster are recruited/identified, exposed and assessed in one, long, uninterrupted stream. This is known as a continuous recruitment design.^11,12^ The standard approach to the design of these stepped wedge trials is to divide time into periods of equal length, with cross-overs at the boundaries between periods. With time on a continuous scale, however, there are no canonical cross-over times, and the number of sequences is limited only by the total number of clusters: each cluster could cross from the control to the intervention at any point on the continuous time-scale.

While this means that designs in continuous time can become quite complicated,^13,14^ there may be practical advantages, from the point of view of trial conduct, in choosing a design that has some parsimony, symmetry, or other simplicity of form. In this article, we are motivated in particular by an interest in designing continuous recruitment stepped wedge trials with small numbers of randomised sequences. We keep this investigation simple by concentrating on designs with just three sequences. (Previous work has considered the case of a two-sequence design where one sequence remains in the control condition throughout.)^15^ Aside from their simplicity, designs with small numbers of sequences also make it easier to balance the randomisation according to cluster characteristics.

The problem of optimal design in this case is quite different to the problem of designing an optimal stepped wedge trial with discrete time periods and an equal number of participants in each period.^4–9^ In the continuous recruitment design problem, we can effectively move the cross-over time in each sequence with a slider control: by moving it to the right on the time axis we steadily increase the number of control participants in each cluster in that sequence, but at the cost of steadily decreasing the number of intervention participants by a corresponding amount. A continuous time-scale also allows us to model an intra-cluster correlation (ICC) that varies smoothly as a function of separation in time: the further apart we recruit two participants from the same cluster, the weaker we might expect the correlation between their outcomes to be.^12^

**Figure 1.**
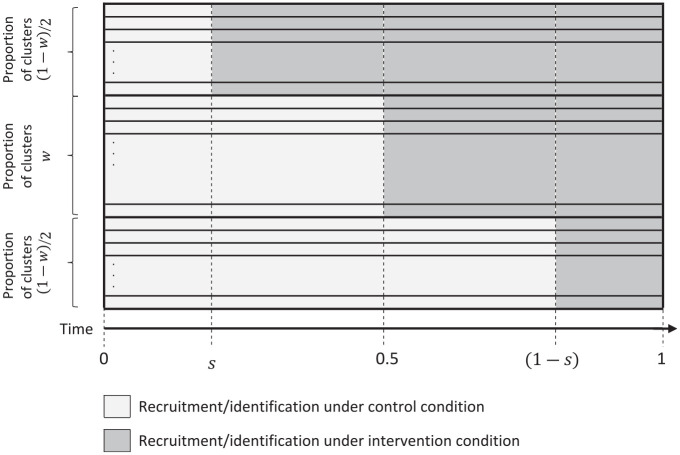
Schematic for a centrosymmetric, three-sequence stepped wedge trial with continuous recruitment. Centrosymmetry is the property that if we run time backwards, and swap intervention and control, then we arrive at the same design.

Three-sequence designs are relatively simple to characterise, particularly if we focus attention on designs that have *centrosymmetry*. A centrosymmetric design has the property that if we run time backwards, and swap intervention and control, then we arrive at the same design.^16,17^ Given the number of clusters, duration of the recruitment period, and rate of recruitment at each cluster, the things that we can control in a centrosymmetric, three-sequence stepped wedge trial are (a) the proportion of clusters allocated to the middle sequence, w , and (b) the timing of the first cross-over, s (Figure 1).

Here, we investigate the choice of design that minimises the variance of the intervention effect estimator under different assumptions about the correlation of outcomes within the same cluster and consider whether there might be simple design choices that are robustly efficient in this sense, even when there is a degree of uncertainty about these unknown correlation parameters. The lower the variance of the intervention effect estimator, the higher the power to detect a given intervention effect at a certain significance level.

We implicitly assume a large number of clusters are available to be randomised. We use generalised least squares methods and asymptotic approximations to calculate the variance of the intervention effect estimator, and we assume, whatever the number of clusters, that we can allocate them to different sequences in whatever allocation ratios we happen to be discussing. A surprisingly high proportion of published stepped wedge trials are conducted with fewer than 10 clusters (33% according to one review).^18^ Designing stepped wedge trials with very small numbers of clusters goes against most methodological guidance,^19,20^ but for practical reasons, we can expect many stepped wedge trials to include only moderate numbers of clusters. We include simulations for one such trial design scenario to illustrate how empirical statistical power matches the power derived from our large-sample formula in this case.

### Motivating example

PATHWEIGH is a weight loss intervention for use in a primary care setting that uses tools built into the electronic medical record to improve workflow and address various barriers to prioritising weight management.^21^ Suresh and colleagues published a protocol for a stepped wedge trial of PATHWEIGH where the clusters are 57 family and internal medicine clinics in a large health system in Colorado, USA.^21^ Patients are identified over a 4-year period beginning 17 March 2020 and are eligible to be included in the trial if they are aged 18 years or over and overweight (body mass index (BMI) ≥25 kg/m^2^) at an initial, index visit.

The investigators estimate that a minimum of 30 patients per clinic per year will be identified. The primary outcome measure is weight loss 6 months after the index visit, extracted from the electronic medical record. PATHWEIGH, being an electronic intervention, can be ‘turned on’ at a clinic at any time, and a patient’s intervention status is defined according to whether the clinic they attend was in the control (routine care) condition or intervention condition at the patient’s index visit.

The trial randomises clinics 1:1:1 to three sequences, in which the intervention is turned on after 1 year, 2 years, or 3 years, respectively. In terms of the design parameters of a centrosymmetric, three-sequence stepped wedge design, the timing of the first cross-over, s=1/4 and the proportion of clusters allocated to the middle sequence, w=1/3 in this case. This kind of regular spacing is traditional in stepped wedge designs. But what if we were willing to consider alternative allocation ratios, or alternative timings for the first and last cross-overs in a three-sequence design? Is there a more efficient choice of s and w ?

## Methods

### Statistical model

To describe the general scenario that we consider, we suppose that time is re-scaled so that the recruitment/identification period runs from time 0.0 to time 1.0. For simplicity, we assume that in each cluster we recruit the same number of individuals, m , over the course of this unit time interval.

We assume a continuous outcome $Y_{\mathrm{ij}}$ for individual i=1,…,m from cluster j=1,…,J , with

(1) $$Y_{ij}=\delta .H(t_{ij}-t_{j}^{*})+T\left(t_{ij},\beta \right)+\varepsilon_{ij},\varepsilon_{ij}\sim N(0,\sigma^{2})$$

where $t_{\mathrm{ij}}$ is the time at which individual i from cluster j is recruited, $t_{j}^{*}$ is the time at which cluster j crosses over to the intervention, H(x) is a unit step function taking value 0 if x<0 and 1 if x≥0 , and T(t,β) is a function that describes the underlying effect of time on expected outcome (assumed to be the same in all clusters), parameterised by the vector β . Errors $\varepsilon_{\mathrm{ij}}$ are identically distributed but not all independent of one another (see below). In a centrosymmetric, three-step design, all of the cross-over times $t_{j}^{*},j=1\ldots J$ belong to the set {s,0.5,1−s} , for some s with 0≤s<0.5 , and clusters are allocated to these sequences in the respective proportions (1−w)/2 , w and (1−w)/2 , for some w with 0≤w<1 (Figure 1).

The parameter δ is the intervention effect we would like to estimate. Having a single parameter for this intervention effect, expressed through the step function, H , models a situation where the intervention has an instantaneous effect that is then maintained at a constant level and is common to all the clusters.

We assume that $\varepsilon_{\mathrm{ij}}$ and $\varepsilon_{i\prime j\prime }$ are uncorrelated for all j≠j′ , for all i and i′ , that is, outcomes in different clusters are uncorrelated. Within a cluster there may be correlation between outcomes, measured by the ICC. Under a model of exchangeability, this ICC is constant

$$\mathrm{Corr}(\varepsilon_{\mathrm{ij}},\varepsilon_{i\prime j})=\rho \forall i\neq i\prime ,j$$

In this article, we consider a more general model that allows us to investigate what happens if the ICC decays with increasing separation in time^15^

(2) $$\begin{array}{c} \mathrm{Corr}(\varepsilon_{\mathrm{ij}},\varepsilon_{i\prime j})=\rho \tau^{\left|t_{\mathrm{ij}}-t_{i\prime j}\right|}\forall i\neq i\prime ,j\\ \mathrm{Corr}(\varepsilon_{\mathrm{ij}},\varepsilon_{i\prime j\prime })=0\;\forall \;i,i\prime ,j\neq j\prime \end{array}$$

The parameter ρ is the ICC for two participants sampled from the same cluster at the same time (the time-specific ICC). The ICC for two participants sampled at times 1.0 apart is ρτ . The parameter τ is therefore the factor by which the ICC decays over the duration of the recruitment period.

Finally, to make headway with deriving a variance for the treatment effect estimator, we make the simplifying assumption that the times at which individuals are recruited from each cluster are regularly spaced, at intervals 1/m apart, rather than being a random process

(3) $$t_{\mathrm{ij}}=i/m\;\forall \;j$$

### Time effect

The variance of the treatment effect estimator will be adjusted for the time effect, under the assumption that this is correctly specified in the analysis model. Exactly which design minimises this variance would seem to depend on the form of the time effect, T in (1), so how are we to choose this? Here, we consider a time effect that is piecewise constant, with discontinuities that coincide with the cross-over times. That is

(4) $$\begin{array}{cc} T\left(t,\beta_{1},\beta_{2},\beta_{3},\beta_{4}\right) & =\beta_{1}+\beta_{2}H\left(t-s\right)+\beta_{3}H\left(t-0.5\right)\\ +\beta_{4}H\left(t-1+s\right) \end{array}$$

While it might seem strange at first sight to choose a model for the time effect that depends on the design, we make this choice for a number of related reasons. First, the regularly spaced recruitment times at each cluster, i/m,i=1,…,m , and the fact that the cross-over times align perfectly with the discontinuities in an otherwise constant time effect, mean that the variance of the treatment effect estimator under the four-parameter functional form for T in (4) is identical to the variance if we were to assume a completely general time effect that was different at each of the times i/m,i=1,…,m . (This follows from a more general invariance result proved elsewhere.)^22^ Second, experience with two-sequence designs suggests that the optimal design solution under (4) may also be a reasonable approximation to the solution under other models with a smooth time effect, such as a polynomial function of sufficiently high degree.^15^ Third (and more qualitatively), adjusting for an underlying time effect that has step-changes at exactly the times the intervention is switched on in certain clusters feels like it ought to offer a ‘strong’ test of a genuine intervention effect.

In fact, if we were faced with a real-life dataset with m=100 , say, it is unlikely that we would use as many as 100 parameters for modelling the effect of time, or that we would assume the time effect was completely smooth. It is more likely that we would allow for step-changes at the cross-overs, and otherwise model time in some parsimonious but plausible way that fitted the data and our prior beliefs. As long as this analysis model for the time effect is correctly specified, then the invariance result mentioned above shows that the variance of the treatment effect estimator will be the same as the variance calculated to hold under model (4).^22^

### Variance of the treatment effect estimator

If we write outcomes $Y_{\mathrm{ij}}$ as a single column vector, Y , and rewrite equations (1)–(4) as

$$Y=X\left(\begin{array}{c} \delta \\ \beta \end{array}\right)+\varepsilon ,\varepsilon \sim N\left(0,V\right)$$

Then, the generalised least squares estimator for the parameters is obtained as

$$\left(\begin{array}{c} \hat{\delta }\\ \hat{\beta } \end{array}\right)={\left(X\prime V^{-1}X\right)}^{-1}X\prime V^{-1}Y$$

and the variance of $\hat{\delta }$ is the first diagonal element of the variance-covariance matrix given by

(5) $$\mathrm{Var}\left(\begin{array}{c} \hat{\delta }\\ \hat{\beta } \end{array}\right)={\left(X\prime V^{-1}X\right)}^{-1}$$

The results presented in this article were obtained with the help of numerical matrix inversion, making use of the fact that the matrix V is block diagonal, owing to the independence of outcomes in different clusters. We used Mata, the matrix programming language within Stata, which is a C-style language that is compiled to produce fast-running code (StataCorp, College Station, Texas, USA). Our code can be accessed online (https://github.com/richard-hooper/SW-3sequence-continuous-recruitment).

### Scenarios

Previous work on optimal stepped wedge design suggests, in the case τ=1 at least, that the optimal design choice depends on the total number of participants in each cluster, m , and time-specific ICC, ρ , via the single parameter mρ/(1−ρ) .^4,5^ This parameter is key because it determines the correlation between mean outcomes in within-cluster comparisons. If clusters are randomised to a sequence that crosses over from the control to the intervention condition half-way through the recruitment period, for example, then the correlation between the mean outcome in a cluster before cross-over ( m/2 participants) and the mean outcome in that cluster after cross-over ( m/2 participants) is given by the following expression, which is a function of R:=mρ/(1−ρ) ^23^

$$\frac{\frac{m}{2}\rho }{1+\left(\frac{m}{2}-1\right)\rho }=\frac{R}{2+R}$$

For given τ<1 , we hypothesise a similar role for the parameter mρ/(1−ρ) in determining the optimal design. Previous work on two-sequence designs with continuous recruitment proposed design rules of thumb based on the product mρ .^15^ Since the parameter mρ is somewhat simpler, and for small ρ the parameters mρ and mρ/(1−ρ) are almost identical, we consider a range of values for m and mρ . In order to limit the set of parameter values and to follow a systematic approach, we consider m∈{50,200,1,000} and, for each m , values of ρ for which mρ∈{0.2,0.5,1,2,5,10,20} . These scenarios are investigated for τ∈{1.0,0.5,0.1} . By presenting results for fixed mρ side by side, we can see how closely the design recommendations match for different m . In the online supplement, we also show what results look like when we keep mρ/(1−ρ) fixed for different m , to confirm the intuition that this is the parameter that strictly determines the optimal design.

### Contour plots

We transform the variance of the treatment effect estimator to a log scale, and draw contour plots of the log-variance over the design parameter space 0≤s<0.5 and 0≤w<1 , for each of the scenarios considered. Contour lines are separated by log(1.1), so that moving from one contour to the next represents a 10% increase in the variance. The lowest contour value is set at the minimum of the log-variance surface. Thus, the innermost contour is the region where the variance is within 10% of the minimum attainable. Because we are particularly interested in regions of low variance, and to reduce the amount of ink used, we have not drawn all of the higher variance contour lines. The variance of the treatment effect estimator will be a multiple of $\sigma^{2}/J$ , $\sigma^{2}$ being the variance of each outcome $Y_{\mathrm{ij}}$ , and J being the number of clusters. Hence, the appearance of the contour plot as described does not depend on either $\sigma^{2}$ or J .

## Results

**Figure 2.**
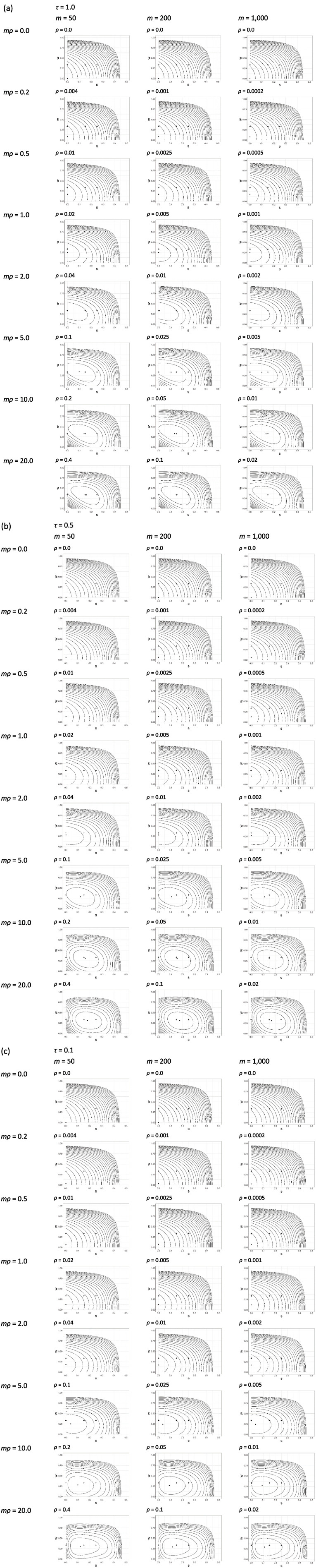
Contour plots of the log of the variance of the treatment effect estimator for different mr and m, where m is the recruitment rate at a cluster, and r is the intra-cluster correlation. Time is scaled from 0 to 1 over the recruitment period. Contour plots are drawn over the design parameter space 0≤*s*<0:5 and 0≤*w*<1, where s is the first cross-over time and w is the proportion of clusters allocated to the middle sequence. The solutions *s*=0, *w*=1/3, *s*=0:15, *w*=1/3, and *s*=0:25, *w*=1/3 (see text) are marked with a ‘+’ symbol. Contour lines are separated by log(1.1), so that moving from one contour to the next represents a 10% increase in the variance. The lowest contour value is set at the minimum of the log-variance surface (the small, circular mark on each plot marks this minimum). The factor, τ, by which the intra-cluster correlation decays over unit time is (a) *τ*=1:0; (b) *τ*=0:5; (c) *τ*=0:1.

Contour plots for τ=1.0 , τ=0.5 , and τ=0.1 are shown in Figure 2(a)–(c). For given τ and mρ , the plots for different m are remarkably similar, confirming that the shape of the variance surface, and therefore the choice of optimal design, depends on m and ρ primarily through their product. The greatest divergence between the plots in a given panel of these figures is for τ=0.1 , mρ=20.0 (bottom row of Figure 2(c)). Supplemental Figure 1 (in the online supplement) shows contour plots for τ=0.1 , mρ/(1−ρ)=20 , m∈{50,200,1,000} . These three plots are almost identical, illustrating that the dependence is strictly via mρ/(1−ρ) . To present a relatively simple overview of our results below, with intuitive rules of thumb, we will continue to talk in terms of mρ .

When mρ is zero, the optimal design gives zero weight to the middle sequence and puts the first cross-over at time zero. If mρ is zero, then observations within clusters are independent, so this design – a two-sequence, parallel groups design with one sequence in the intervention condition throughout and the other in the control condition throughout (analogous to an individually randomised parallel groups design) – is exactly what we would expect to see. As mρ starts to increase from 0.0, the optimal weight for the middle sequence increases, but the optimal time for the first cross-over remains at zero. This means there are clusters that remain in the control condition or the intervention condition for the entire trial. In the world of discrete-period stepped wedge designs, these are known as hybrid designs, because they are a hybrid of a parallel groups design and a classic stepped wedge design where all the clusters cross over at some point.^4^ Somewhere between mρ=2.0 and mρ=5.0 (at least for τ≥0.1 ), the optimal time for the first cross-over breaks away from zero. The shape of the plotted surface depends on τ , although there are qualitative similarities between plots for given mρ and different τ .

The variance surfaces in the plots have relatively flat bottoms, suggesting, first, that for any given mρ and τ there may be a close-to-optimal solution where s and w are simple fractions, and second, that it may be possible to find a solution that is close-to-optimal over a range of values for mρ and τ , thus accommodating some uncertainty in these parameters. Look at the solution with s=0 , w=1/3 , for example, which is one of the solutions marked with a ‘+’ in Figure 2. This simple, hybrid design allocates clusters in equal proportions to three sequences. For any mρ in the range 0.2–5.0 and any τ between 0.1 and 1.0, this design falls within the innermost contour of the plot, that is, the region where the variance is within 10% of the minimum attainable, suggesting it could be an acceptable design in all these scenarios. Or consider the solution with s=0.15 , w=1/3 , which is the second solution marked with a ‘+’. This is perhaps not a ‘natural’ choice of design, but nevertheless falls within the innermost contour of the plot for any mρ of 5.0 or greater and any τ between 0.1 and 1.0, making it a robustly efficient choice in this broad range of cases.

A design with equally spaced steps and equally weighted sequences ( s=0.25 , w=1/3 ), on the other hand, which is the third solution marked in Figure 2 with a ‘+’, is borderline at best (at least in the scenarios plotted), achieving its most favourable performance when mρ=20 and τ=0.5 .

### Design and sample size in the motivating example

In our motivating example, the PATHWEIGH trial, the assumption was that 30 patients would be recruited per year from each clinic over 4 years, so that m=120 . The power calculation for PATHWEIGH assumed an ICC somewhere between 0.02 and 0.05. If we take this to reflect an interval estimate of the time-specific ICC, ρ , then we want a design that is robustly efficient with mρ in the range 2.4–6.0. The PATHWEIGH power calculation did not discuss the decay in the ICC, but let us suppose, for the sake of argument, that we want to allow for a decay of up to 0.5 in the ICC over the 4-year trial period, so that 0.5≤τ≤1 . We should remember that a prior estimate of the decay in the ICC over 4 years may be much harder to come up with than a prior estimate of the time-specific ICC. (Of course, when presented with an estimate of the ICC that does not mention the decay over time at all, as in the PATHWEIGH example, there is a question as to whether this is really an estimate of the time-specific ICC, or some average of the decaying ICC over the entire recruitment period.^24^ This speaks to the importance of specifying assumptions about the ICC when reporting sample size calculations for stepped wedge trials.)^25^

Looking at our contour plots, we might then choose s intermediate between 0 and 0.15 – s=1/12 , say – and w=1/3 , as a solution that is robustly efficient. In this design, the intervention is switched on after 4 months, 2 years, and 3 years and 8 months in the respective sequences.

Recall from the Methods that the variance of the treatment effect estimator is a multiple of $\sigma^{2}/J$ , where $\sigma^{2}$ is the variance of each outcome, and J is the number of clusters. Let us call this $\theta \sigma^{2}/J$ . To calculate the number of clusters needed to demonstrate an effect $\delta^{*}$ with power 1−β at the 5% significance level, we can therefore apply the ‘fundamental equation’ of sample size^26^

(6) $$J\geq {\left(z_{0.975}+z_{1-\beta }\right)}^{2}{\left(\frac{\sigma }{\delta^{*}}\right)}^{2}\theta$$

where $z_{p}$ is the $p^{\mathrm{th}}$ percentile of the standard normal distribution.

Table 1 shows the variance of the treatment effect estimator for the design s=1/12 , w=1/3 , at the four corners of the selected parameter range for mρ and τ . The variance is calculated from the matrix expression in the Methods. Table 1 also shows the total number of clusters (allocated equally to the three sequences) required to detect a mean difference in weight loss of 1 kg, 1.25 kg, or 1.5 kg with 80% power at the 5% significance level, assuming (as the PATHWEIGH investigators did) that the standard deviation of weight loss is 10.7 kg. The number of clusters is rounded up to the next multiple of three. For comparison, Table 1 shows the same requirements for the ‘standard’ stepped wedge design (the design adopted for PATHWEIGH) with s=0.25 , w=1/3 . The non-standard design always out-performs the standard one – in some scenarios, considerably so.

**Table 1.** Total number of clusters needed in the PATHWEIGH example (see text) to achieve 80% power at the 5% significance level to detect different treatment effects, under different scenarios concerning correlations between outcomes from the same cluster. *J* is the number of clusters, and σ^2^ is the variance of the outcome.

Scenarios		Non-standard design ( s=1/12,w=1/3 )				Standard design ( s=1/4,w=1/3 )			
		Variance of the treatment effect estimator	Number of clusters needed to detect a difference in mean weight loss of			Variance of the treatment effect estimator	Number of clusters needed to detect a difference in mean weight loss of		
ρ	τ		1 kg	1.25 kg	1.5 kg		1 kg	1.25 kg	1.5 kg
0.02	1.0	0.0793 $\sigma^{2}/J$	72	48	33	0.1002 $\sigma^{2}/J$	93	60	42
0.02	0.5	0.0820 $\sigma^{2}/J$	75	48	33	0.1054 $\sigma^{2}/J$	96	63	45
0.05	1.0	0.0928 $\sigma^{2}/J$	84	54	39	0.1054 $\sigma^{2}/J$	96	63	45
0.05	0.5	0.1093 $\sigma^{2}/J$	99	63	45	0.1217 $\sigma^{2}/J$	111	72	51

The theoretical power (from equations (5) and (6)) of each of the designs presented in Table 1 was compared with the empirical power estimated by simulation. For each scenario and design, 1,000 replicated datasets were generated in R, and the continuous time decay model in equations (1) to (4) was fitted using the glmmTMB package in R.^27^ This analysis did not include any small-sample correction to mitigate inflation of the nominal Type I error rate and power that might result from the moderate number of clusters. Such corrections are becoming more widely available in software implementations for the analysis of cluster randomised trials,^28^ but also add considerably to the processing time, which can make large-scale simulations challenging. Our code can be accessed online (https://github.com/richard-hooper/SW-3sequence-continuous-recruitment). Supplemental Figure 2 displays the results in a nested loop plot. Empirical power closely matched theoretical power when the time-specific ICC was 0.05. Empirical power was inflated when the time-specific ICC was 0.02, and this was particularly evident with the non-standard design. The performance of the glmmTMB package (and alternatives) for analysing data from longitudinal cluster randomised trials with a continuous time decay model for the ICC warrants further investigation.

## Conclusion

We have illustrated optimal designs for three-sequence stepped wedge trials with continuous recruitment, under different assumptions about the correlation of outcomes from the same cluster. We conclude that under given assumptions there may be a relatively simple design that offers close-to-optimal efficiency and that there may be designs that are robustly efficient over a wide range of assumptions. If efficiency is offered as one of the justifications for using a stepped wedge design over a parallel groups design, then we should design with optimal efficiency in mind. The focus of this article has been on design, informed by theory. Suitable approaches to analysis that can handle a continuous time model (including the decay in the ICC) and also control the Type I error rate when the number of clusters is moderate or small need further evaluation and comparison.

The way in which the ICC changes over time matters to the design, and it is important to articulate these assumptions when reporting sample size calculations for stepped wedge trials.^25^ We assumed a particular parametric form for the decay in the ICC to help us understand the more general impact of this kind of decay on optimal design. Other models for the ICC could, of course, be investigated. As in earlier work,^15^ we simplified considerably in assuming that eligible participants present at regular, fixed intervals rather than as a random continuous-time process, but assuming that the arrival rate is constant over time we would expect arrival times in a sample to become increasingly uniformly spread as the rate increases. Simulation studies that have investigated the impact of unevenly spaced arrival times on precision of the treatment effect estimator in the context of stepped wedge designs suggest that this impact is small.^29^

Our focus has been on the optimal design of three-sequence trials, but our findings may also offer clues about optimal design with larger numbers of sequences. With more sequences, there are more degrees of freedom to the design space for centrosymmetric designs, which becomes correspondingly harder to visualise and requires more effort to search exhaustively. Nevertheless, previous work on optimal design in the discrete time case, with no decay in the ICC, has shown that the ‘internal’ sequences (i.e. sequences other than the first and last) should all be given equal weight.^4,5^ There may be similar simplifications when we move over to considering designs with many sequences in the continuous time setting. Ultimately, however, we may prefer a design with fewer sequences for its greater simplicity and practicality.

## Supplemental Material

sj-docx-1-ctj-10.1177_17407745241251780 – Supplemental material for Efficient designs for three-sequence stepped wedge trials with continuous recruitment Supplemental material, sj-docx-1-ctj-10.1177_17407745241251780 for Efficient designs for three-sequence stepped wedge trials with continuous recruitment by Richard Hooper, Olivier Quintin and Jessica Kasza in Clinical Trials
