# Supplementary material for: Efficient designs for three-sequence stepped wedge trials with continuous recruitment
Source: Clin Trials. 2024 May 21;21(6):723–33. doi: 10.1177/17407745241251780 (PMC11528865; doi:10.1177/17407745241251780)

*Appendix to the article*

**Efficient designs for three-sequence stepped wedge trials with continuous recruitment**

Richard Hooper^1^, Olivier Quintin^1^, Jessica Kasza^2^

^1^ Wolfson Institute of Population Health, Queen Mary University of London, London, UK

^2^ School of Public Health and Preventive Medicine, Monash University, Melbourne, Australia

**Supplemental Figure 1**. Contour plots of the log of the variance of the treatment effect estimator for recruitment rate at a cluster, $m\in\left\{ 50, 200, 1,000 \right\}$, time-specific intra-cluster correlation, $\rho$, calculated so that $m\rho/(1-\rho)=20.0$, and decay in the intra-cluster correlation over the recruitment period, $\tau=0.1$. Time is scaled from 0 to 1 over the recruitment period. Contour plots are drawn over the design parameter space $0\leq s<0.5$ and $0\leq w<1$, where $s$ is the first cross-over time and $w$ is the proportion of clusters allocated to the middle sequence. The solutions $s=0$, $w=1/3$, $s=0.15$, $w=1/3$, and $s=0.25$, $w=1/3$ (see article text) are marked with a “+” symbol. Contour lines are separated by log(1.1), so that moving from one contour to the next represents a 10% increase in the variance. The lowest contour value is set at the minimum of the log-variance surface (the small, circular mark on each plot marks this minimum).

| *m* = 50 | *m* = 200 | *m* = 1,000 |
| --- | --- | --- |
|  |  |  |
| *ρ* = 0.2857 | *ρ* = 0.0909 | *ρ* = 0.0196 |
|  |  |  |
| 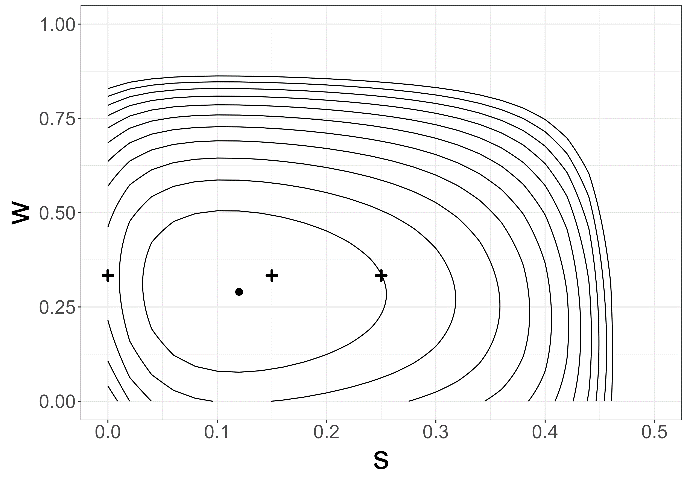 | 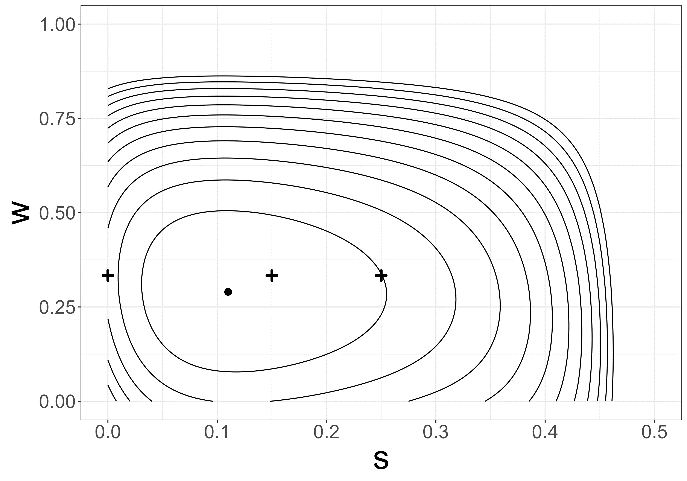 | 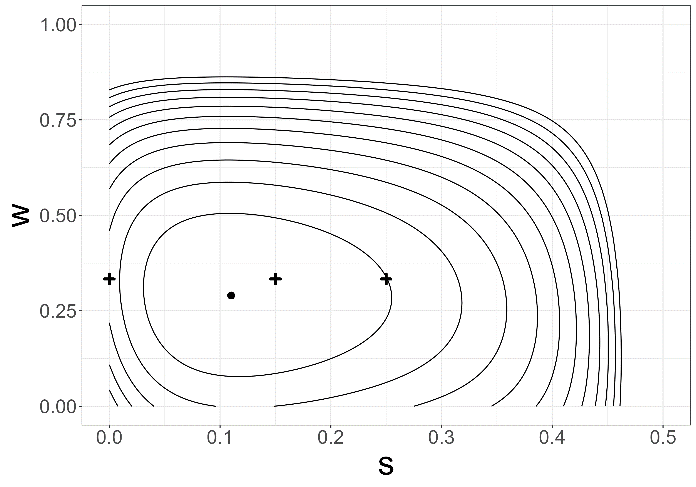 |

**Supplemental Figure 2**. Empirical and theoretical power for the simulation study covering the scenarios considered in the PATHWEIGH example. Empirical power is estimated by simulation (plus or minus 2.0 Monte Carlo standard errors, based on 1,000 replications); theoretical power is based on large-sample theory. Results for the non-standard design appear in the top panel; results for the standard design in the bottom panel. For each design, the theoretical and empirical power are displayed for each combination of: difference to be detected (1 kg, 1.25kg, 1.5kg); τ=0.5,1.0; and time-specific ICC = 0.02, 0.05.


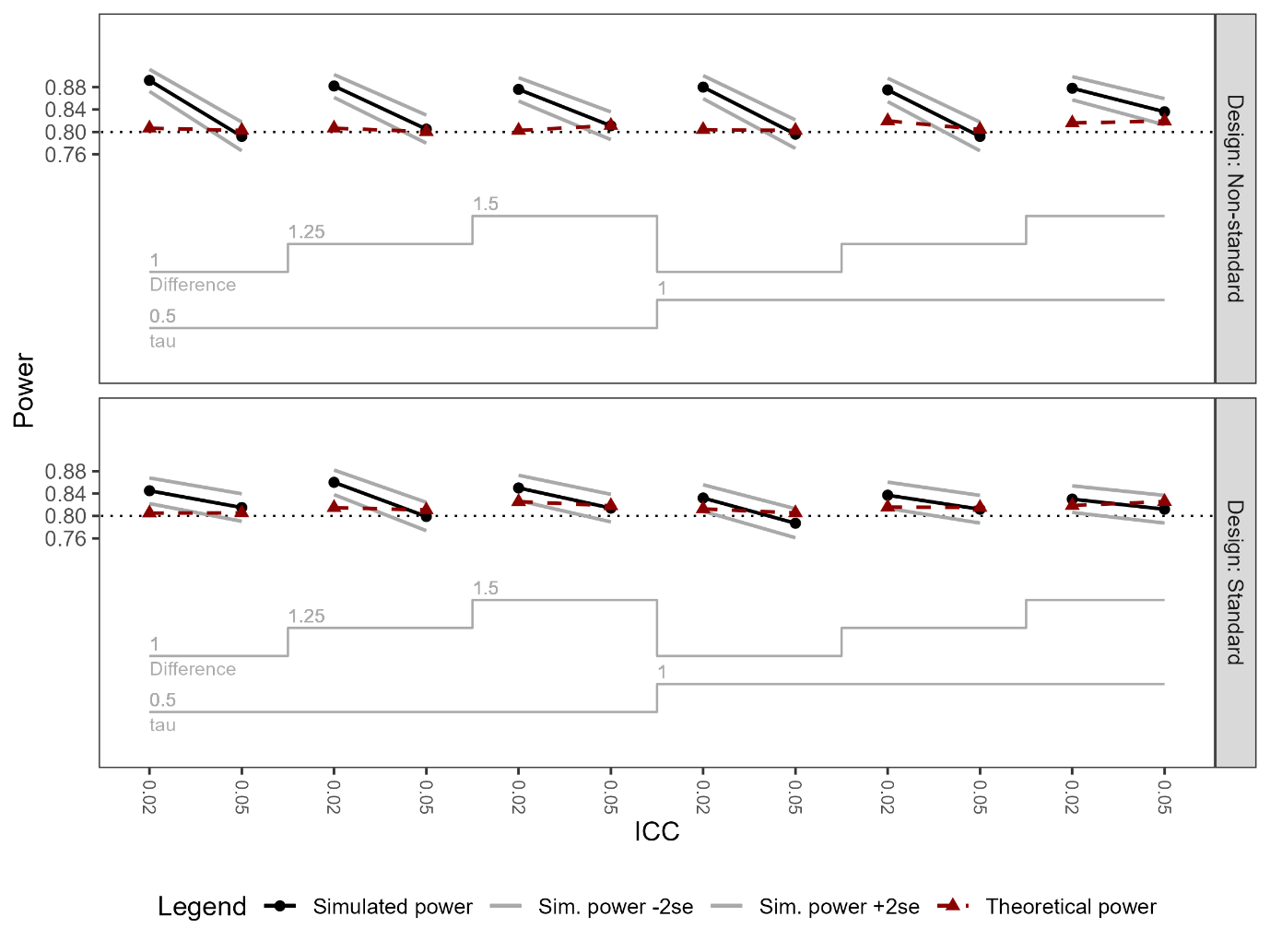

Supplement: sj-docx-1-ctj-10.1177_17407745241251780 – Supplemental material for Efficient designs for three-sequence stepped wedge trials with continuous recruitment [file sj-docx-1-ctj-10.1177_17407745241251780.docx]
